# Molecular Detection of *Histoplasma* in Bat-Inhabited Tunnels of Camino de Hierro Tourist Route, Spain

**DOI:** 10.3201/eid3105.241117

**Published:** 2025-05

**Authors:** Joaquina María García-Martín, Julio David Soto López, Diego Lizana-Ciudad, Pedro Fernández-Soto, Antonio Muro

**Affiliations:** Universidad de Salamanca, Salamanca, Spain (J.M. García-Martín, J.D. Soto López, D. Lizana-Ciudad, P. Fernández-Soto, A. Muro); Instituto de Investigación Biomédica de Salamanca, Salamanca (J.M. García-Martín, J.D. Soto López, P. Fernández-Soto, A. Muro); Centro de Estudios Ambientales y Dinamización Rural, Salamanca (D. Lizana-Ciudad)

**Keywords:** fungi, zoonoses, histoplasmosis, Histoplasma, bats, Camino de Hierro, environmental detection, fungal pathogen, guano, systemic mycoses, thermally dimorphic fungi, zoonotic risk, Spain

## Abstract

We detected *Histoplasma capsulatum* in 2 bat-inhabited tunnels of a tourist route in northern Spain. This finding confirms that the geographic distribution of this fungal pathogen is wider than previously thought. Our results highlight the need for surveillance and assessment of the potential infection risk for workers and visitors.

Histoplasmosis is a primary pulmonary infection caused by inhalation of aerosolized spores of *Histoplasma* fungi, naturally present in soils, after disruption of soil aggregates (*1*). This fungal disease is endemic to tropical, subtropical, and temperate regions, and most cases occur in Latin America (e.g., Brazil, Guyanas), North America (mainly in the Ohio and Mississippi River Valleys), and Western and Central Africa. Most cases occurring in Latin America are attributed to *H. capsulatum* sensu stricto (formerly *H. capsulatum* Panama or H81 lineage) and *H. suramericanum* (formerly *H. capsulatum* LAm A lineage). In North America, *H*. *mississippiense* (formerly *H. capsulatum* NAm 1) and *H*. *ohiense* (formerly *H. capsulatum* NAm 2) are the predominant etiologic agents of chronic pulmonary histoplasmosis; *H*. *ohiense* is more virulent (*2*). Cases in Africa, often with skin and bone involvement, are associated with *Histoplasma* varietas *duboisii*, also referred to as *H. duboisii* or *H. capsulatum* H88 lineage (*2*).

Different species of the genus *Histoplasma* are found in soils enriched with bird and bat guano, which contribute to the accumulation of nitrogen and phosphorus in soils, favoring fungal growth. In particular, bats (order Chiroptera) act as natural reservoirs and dispersers of *Histoplasma*, which is often isolated from their organs and guano collected from natural and artificial roosts, including abandoned caves and tunnels, mines, cellars, or basements (*3*). In addition, numerous histoplasmosis outbreaks linked to bat guano exposure have been reported; for example, a severe histoplasmosis outbreak with fatal outcomes occurred among workers in abandoned tunnels contaminated with spore-bearing dust in the Dominican Republic (*4*).

In nonendemic regions, such as Europe, few imported cases of histoplasmosis and even fewer apparently autochthonous cases have been reported (*5*). Regarding the presence of *Histoplasma* spp. in the environment, reports are related to isolations by traditional culture methods from guano and soil samples collected in caves inhabited by bats in Romania and in a chicken farm in Italy (*6*,*7*). In addition, in northern Italy, some persons tested positive for histoplasmin skin tests, suggesting that environmental conditions may have enabled establishment and spread of *Histoplasma* in certain areas of Europe (*8*). In Spain, *Histoplasma* has not been isolated from bat organs or guano, and most clinical cases of histoplasmosis have been considered imported, resulting from international travel and migration (*9*). Numerous cases of histoplasmosis related to bat exposure have been reported in Africa (*10*), Central America (*4*), South America (*11*), and North America (*12*). We describe detection of *H. capsulatum* in 2 bat-inhabited tunnels of a tourist route in northern Spain.

## The Study

To shed light on the distribution of *Histoplasma* beyond traditional known endemic areas, we conducted a study in 2 bat-inhabited tunnels of the Camino de Hierro in Salamanca, northern Spain, a pedestrian route receiving >60,000 visitors since its opening as an ecotourism attraction in 2021 (Appendix). We collected 101 guano samples in the tunnels (Appendix Table 1) and found almost 42% were positive for *Histoplasma* by nested PCR, using previously published primers (Appendix Table 2). Specifically, the *Hcp100* gene sequences we isolated (submitted to GenBank under accession nos. PP887829–78) (Table) shared high homology with GenBank sequences corresponding to *H*. *capsulatum* s.s., *H. suramericanum*, *H. capsulatum* LAm B2, and *H*. *capsulatum* var. *duboisii*. Moreover, our phylogenetic analyses (Appendix) indicated that the newly obtained sequences form a fully supported monophyletic group with multiple GenBank sequences of *Histoplasma* (posterior probability = 1; bootstrap support = 100%), without a clear geographic or host-related pattern (Figure).

**Table:** Identities of *Histoplasma* isolates obtained from guano samples collected in the tunnels of the Camino de Hierro tourist route in study of molecular detection of *Histoplasma*, Spain*

Isolate code	Isolate BLAST identity (sublineage)†	Isolate accession no.	Source species for guano samples	Source accession no.
H1	*H. suramericanum* (LAm A1)	PP887829	*Rhinolophus ferrumequinum*	PP919660
H2	*H. suramericanum* (LAm A1)	PP887830	*Myotis blythii*	PP919661
H3	*H. suramericanum* (LAm A1)	PP887831	*Myotis* sp.	PP919662
H4	*H. capsulatum* variant *duboisii/H. capsulatum* sensu lato (LAm B2)	PP887832	*M. blythii*	PP919663
H5	*H. suramericanum* (LAm A1)	PP887833	*Myotis* sp.	PP919664
H6	*H. capsulatum* var. *duboisii/H. capsulatum* s.l. (LAm B2)	PP887834	*M. blythii*	PP919665
H7	*H. capsulatum* var. *duboisii/H. capsulatum* s.l. (LAm B2)	PP887835	*M. blythii*	PP919666
H8	*H. capsulatum* var. *duboisii/H. capsulatum* s.l. (LAm B2)	PP887836	*Myotis* sp.	PP919667
H9	*H. suramericanum* (LAm A1)	PP887837	*M. blythii*	PP919668
H10	*H. suramericanum* (LAm A1)	PP887838	NA	NA
H11	*H. capsulatum* var. *duboisii/H. capsulatum* s.l. (LAm B2)	PP887839	*Miniopterus schreibersii*	PP919669
H12	*H. suramericanum* (LAm A1)	PP887840	*M. blythii*	PP919670
H13	*H. suramericanum* (LAm A1)	PP887841	*M. blythii*	PP919671
H14	*H. suramericanum* (LAm A1)	PP887842	*M. blythii*	PP919672
H15	*H. suramericanum* (LAm A1)	PP887843	*M. blythii*	PP919673
H16	*H. suramericanum* (LAm A1)	PP887844	*M. blythii*	PP919674
H17	*H. capsulatum* var. *duboisii/H. capsulatum* s.l. (LAm B2)	PP887845	*Myotis* sp.	PP919675
H18	*H. suramericanum* (LAm A1)	PP887846	*M. blythii*	PP919676
H19	*H. suramericanum* (LAm A1)	PP887847	*M. blythii*	PP919677
H21	*H. suramericanum* (LAm A1)	PP887849	*Myotis* sp.	PP919679
H22	*H. suramericanum* (LAm A1)	PP887850	*Myotis* sp.	PP919680
H23	*H. suramericanum* (LAm A1)	PP887851	*M. blythii*	PP919681
H26	*H. suramericanum* (LAm A1)	PP887854	*Myotis* sp.	PP919684
H27	*H. suramericanum* (LAm A1)	PP887855	*M. blythii*	PP919685
H28	*H. suramericanum* (LAm A1)	PP887856	*M. blythii*	PP919686
H29	*H. suramericanum* (LAm A1)	PP887857	*R. ferrumequinum*	PP919687
H30	*H. capsulatum* var. *duboisii/H. capsulatum* s.l. (LAm B2)	PP887858	*M. blythii*	PP919688
H31	*H. suramericanum* (LAm A1)	PP887859	NA	NA
H32	*H. suramericanum* (LAm A1)	PP887860	*M. blythii*	PP919689
H33	*H. suramericanum* (LAm A1)	PP887861	*M. blythii*	PP919690
H34	*H. capsulatum* var. *duboisii/H. capsulatum* s.l. (LAm B2)	PP887862	*Myotis* sp.	PP919691
H35	*H. capsulatum* var. *duboisii/H. capsulatum* s.l. (LAm B2)	PP887863	*R. ferrumequinum*	PP919692
H36	*H. suramericanum* (LAm A1)	PP887864	*Myotis* sp.	PP919693
H37	*H. capsulatum* sensu stricto	PP887865	*M. blythii*	PP919694
H38	*H. capsulatum* var. *duboisii/H. capsulatum* s.l. (LAm B2)	PP887866	*Myotis* sp.	PP919695
H39	*H. capsulatum* var. *duboisii/H. capsulatum* s.l. (LAm B2)	PP887867	*M. blythii*	PP919696
H40	*H. capsulatum* var. *duboisii/H. capsulatum* s.l. (LAm B2)	PP887868	*M. schreibersii*	PP919697
H41	*H. suramericanum* (LAm A1)	PP887869	*M. blythii*	PP919698
H42	*H. capsulatum* var. *duboisii/H. capsulatum* s.l. (LAm B2)	PP887870	*M. blythii*	PP919699
H43	*H. capsulatum* s.s.	PP887871	*M. blythii*	PP919700
H49	*H. suramericanum* (LAm A1)	PP887877	*M. blythii*	PP919706
H50	*H. suramericanum* (LAm A1)	PP887878	*M. blythii*	PP919707

*GenBank accession numbers are shown; further details are provided in Appendix Table 4. NA, not available. †Species names, according to (*2*), followed by sublineage when applicable. *H. capsulatum* s.s. = *H. capsulatum* Panama or H81 lineage; *H. capsulatum* var. *duboisii* = *H. capsulatum* African lineage or *H. duboisii*; *H. suramericanum* = *H. capsulatum* LAm A. Note that the identity of some isolates could not be determined at species level because the corresponding Hcp100 sequences shared high similarity values with several GenBank isolates representing different *Histoplasma* species or lineages (separated by a slash).

**Figure:**
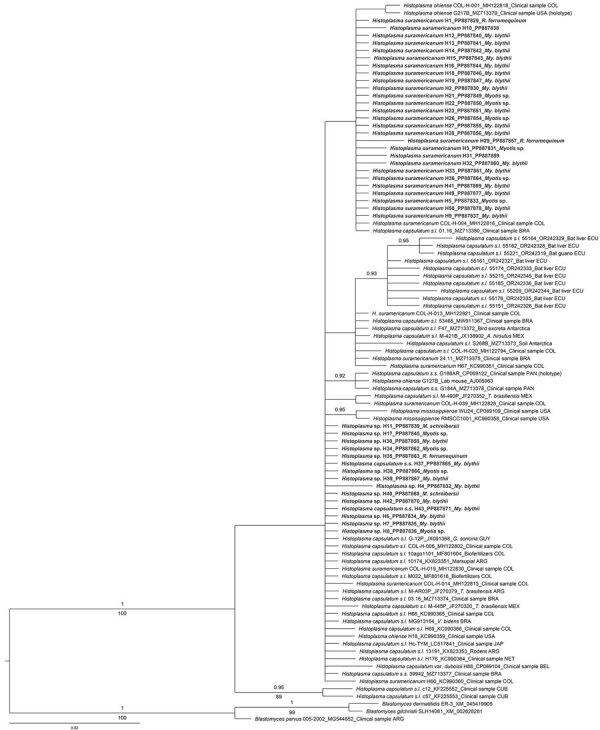
Bayesian tree based on 210-bp partial *Hcp100* gene sequences obtained from 42 guano samples collected in the tunnels of the Camino de Hierro tourist route in study of molecular detection of *Histoplasma*, Spain. Representatives of the genus *Blastomyces* were used as outgroup. GenBank reference isolates are labeled by species name, specimen code, accession number, host or source (if known), and 3-letter country code. Bold text indicates sequences obtained in this study, labeled by taxon name, isolate code, accession number, and source bat species. Numbers above the branches correspond to Bayesian posterior probability and numbers below the branches to maximum-likelihood bootstrap values; values are shown if posterior probability is >0.90 and bootstrap value is >75%. Scale bar indicates average number of substitutions per site.

To determine species of bats roosting in this area, we used BLAST (https://blast.ncbi.nlm.nih.gov) to identify cytochrome c oxidase subunit I sequences obtained from *Histoplasma*-positive samples. Results corresponded to different species that are mainly distributed in Europe: *Myotis blythii* was the most common, followed by *Rhinolophus ferrumequinum* and *Miniopterus schreibersii* (all sequences were submitted to GenBank under accession nos. PP919660–707) (Table). That finding increases the number of possible hosts and dispersers associated with this human pathogen.

The degree of positivity we observed suggests a substantial presence of *Histoplasma* in the tunnels studied, which seems reasonable because the moderate temperature, constant humidity, and darkness of both tunnels investigated are suitable environmental conditions for fungal growth. Of note, outside known areas of endemicity, *Histoplasma* has been isolated from soils contaminated with bat guano in Romania (*6*).

A total of 728 cases of human histoplasmosis have been diagnosed in 17 countries in Europe, of which Spain accounts for up to 60% (*5*). Most cases were imported from Central and South America (*9*), except 4 cases: 1 autochthonous laboratory-acquired case and 3 cases with no epidemiologic history in patients who had never visited endemic areas. One case was in a drug user with HIV/AIDS, another was in a patient who had disseminated histoplasmosis develop after renal transplantation, and the third was in a patient who had occasionally traveled to France, Italy, and the United Kingdom and had previously been treated with an immunosuppressant, suggesting reactivation of a latent infection (*13*). However, to date, *Histoplasma* has not been isolated from environmental samples in Spain, and no autochthonous clinical case of histoplasmosis has been associated with exposure to bats.

## Conclusions

The results of this study indicate that *Histoplasma* is present in bat-inhabited tunnels at Camino de Hierro in northern Spain. That finding evidences that the geographic distribution of this genus is wider than previously thought and also reinforces the known association between *Histoplasma* and bats. 

The risk for histoplasmosis increases with contact with guano deposited in bat roosts. Exploring caves and similar environments is a well-documented source of *Histoplasma* exposure; the first outbreaks of histoplasmosis were related to bat-inhabited locations dating back to the 1930s (*14*). For that reason, the need to assess the presence of *Histoplasma* in bat-inhabited places before opening them to public access has been emphasized (*11*). Therefore, even though no autochthonous cases of histoplasmosis have been reported in Spain, the detection of *Histoplasma* in such a popular tourist attraction as Camino de Hierro makes it advisable to alert local clinicians about the importance of considering histoplasmosis in the differential diagnosis of patients with community-acquired pneumonia. Clinicians can refer to available clinical diagnostic algorithms for histoplasmosis for evidenced-based testing guidance (*15*). 

Our results provide a warning about the presence of *Histoplasma* in Camino de Hierro but also could stimulate further research on bat populations in Spain, opening lines of research into their role in the transmission of histoplasmosis and other airborne infectious diseases. Research on the effects *Histoplasma* and other pathogens on the health of bats should also be considered, and we advise carrying out a serologic study to assess possible exposure to *Histoplasma* among workers and susceptible persons who have visited the tunnels. Given the tourism value and high number of visitors to Camino de Hierro, health authorities should consider our findings and implement measures to prevent potential cases of histoplasmosis, in both visitors and workers of this tourist route. 

Appendix Additional information for molecular detection of *Histoplasma* in bat-inhabited tunnels of Camino de Hierro tourist route, Spain.
