# Supplementary material for: Molecular Detection of Histoplasma in Bat-Inhabited Tunnels of Camino de Hierro Tourist Route, Spain
Source: Emerg Infect Dis. 2025 May;31(5):1010–4. doi: 10.3201/eid3105.241117 (PMC12044256; doi:10.3201/eid3105.241117)
Supplement: Appendix — Additional information for molecular detection of Histoplasma in bat-inhabited tunnels of Camino de Hierro tourist route, Spain. [file 24-1117-Techapp-s1.pdf]

Article DOI: https://doi.org/10.3201/eid3105.241117

EID cannot ensure accessibility for supplementary materials supplied by authors. Readers who have difficulty accessing supplementary content should contact the authors for assistance.

Molecular Detection of *Histoplasma* in Bat-Inhabited Tunnels of Camino de Hierro Tourist Route, Spain

Appendix

Materials and Methods

Sampling location

The study was conducted in Camino de Hierro, a pedestrian route of 17 km (with 20 tunnels and 10 bridges) that runs along a section of a closed railway line built to facilitate the cross-border connection between Spain and Portugal at the end of the 19th century. After almost 40 years of abandonment, the Camino de Hierro was opened as an ecotourism attraction in 2021, receiving since then more than 60,000 visitors (*1*).

The two longest tunnels of Camino de Hierro (tunnel 1, or "La Carretera tunnel," 40°59′08.4"N 6°50′19.5"W, 1500 m long; and tunnel 3, or "Morgado tunnel," 40°57′10.9"N 6°51′06.5"W; 423 m long) are home to one of the most important colonies of hibernating and breeding cave bats in the Iberian Peninsula (Appendix Figure 1).

Only in the tunnel 3, there are more than 12,000 bats of different species (Appendix Figure 2), most listed as vulnerable and/or protected in the Spanish Catalogue of Threatened Species (*2*).

Guano sample collection

A set of 101 guano samples was collected in the tunnels 1 and 3. Samples were collected using sterile scoops from the topsoil under roosting bats after natural defecation, without interacting with the animals at any time to minimize stress (Appendix Figure 1, panel D). They were placed in 50 mL Falcon tubes without preservatives and kept at 4°C during transportation

within 2 h of collection to the laboratory of the Center for Research on Tropical Diseases of the Universidad de Salamanca, CIETUS (Spain).

Sample collection was approved by the Bioethics Committee of the Universidad de Salamanca (ref. RD 53/013, registration no. 965/2023), and authorized by the Servicio Territorial de Medio Ambiente (Delegación Territorial de Salamanca, Junta Castilla y León, Spain), under license AUES_SA_12 (JC)_23.

Genomic DNA extraction

DNA was immediately extracted from guano samples using commercial kits following the manufacturer's instructions, adjusting the elution volume to 50 µL. DNA concentration was quantified using a Nanodrop ND-1000 spectrophotometer (Nanodrop Technologies, United States). DNA extractions were stored at −20°C until use (Appendix Table 1).

PCR amplification and amplicon purification

To detect the presence of *Histoplasma* DNA in guano samples, we amplified by nested PCR a 210-bp fragment of Hcp100, a nuclear gene encoding for a regulatory protein involved in fungal adaptation and survival within macrophages, following methodology previously described (*3*). Each primary reaction mixture contained 12.5 µL of MyTaq Red Mix (Bioline, United Kingdom), 1 µL of each primer (10 nM), 3 µL of DNA template, and Milli-Q water to a final volume of 25 µL, as recommended by the manufacturer. The master mix for nested-PCR reactions was identical, except that 1 µL of the first reaction product was used as DNA template. Additionally, a bat-specific primer pair targeting the mitochondrial cytochrome c oxidase subunit I (COI) gene was used to identify the bat species corresponding to each guano sample positive for *Histoplasma* (Appendix Table 2).

PCR reactions were carried out in a GeneExplorer thermal cycler (Bioer, People’s Republic of China). PCR products were run on 1.5% agarose gels under an UVP Biodoc-It® 2 imaging system (Analytik Jena, Germany) and purified using a Gel and PCR Clean up kit (Macherey-Nagel GmbH & Co. KG, Germany). Purified amplicons were Sanger sequenced in both directions by the Nucleus sequencing service of the Universidad de Salamanca, on a 3500 Genetic Analyzer sequencer (Applied Biosystems, USA), with the same primers used for amplification.

Sequences edition, alignment and phylogenetic analyses

Sequence edition and consensus assembly were performed in Geneious v. 7.1.9 (*4*). After trimming low quality ends, BLAST similarity searches were performed against the NCBI database to identify the source of the sequences. To place our Hcp100 sequences in a phylogenetic context, we downloaded all 301 homologous sequences, obtained from different *Histoplasma* species, available in GenBank on 1st June 2024.

There were many identical sequences so only 51 unique sequences were finally used. Additionally, three sequences generated from representatives of the genus *Blastomyces*, closely related to *Histoplasma* (*5*), were used as outgroup (Appendix Table 3).

The final dataset consisted of 104 sequences, which were automatically aligned with MAFFT (*6*) using the G-INS-I strategy, as implemented in Geneious (*4*).

Maximum likelihood (ML) and Bayesian inference (BI) analyses were conducted. The ML phylogenetic tree was built using IQ-TREE v. 2.2.2.7 (*7*), with the optimal partitioning scheme and the corresponding best-fit model of nucleotide substitution being selected by the integrated version of ModelFinder (*8*). Branch support (BS) was assessed using the "complete bootstrap" option with 1,000 non-parametric replicates.

The BI analysis was carried out using the Metropolis-coupled Markov chain Monte Carlo (MCMCMC) method, as implemented in MrBayes v. 3.2.7a (*9*). To estimate the best-fit substitution model for the single partition identified by ModelFinder, the reversible jumping model choice was used (*10*), allowing a gamma distributed rate heterogeneity across sites, and a proportion of invariant sites. Four independent runs, each with six chains, were executed for 50 million generations, sampling every 1,000, with the first 25% discarded as burn-in and the posterior probabilities (PP) being calculated from the remaining ones. To avoid unnecessary computational overload, the analysis was automatically stopped when the average standard deviation of split frequencies (σ) fell below 0.01.

The convergence of the runs was additionally assessed by checking that the value of the effective sample size (ESS) for each parameter was higher than 200, using Tracer v. 1.7.2 (*11*). Both ML and BI trees were visualized and compared in FigTree v. 1.4.4 (*12*). Significant support was assumed for nodes with BS values ≥70% and PP values ≥0.95. Since both analyses yielded

similar topologies, and no incongruences were detected, only the Bayesian tree showing PP and BS support values is provided in the main text.

Results

Among the 101 DNA samples analyzed, 42 (41.6%) were positive for the Hcp100 gene. BLAST results showed that our sequences share high homology with Hcp100 GenBank sequences corresponding to *H. capsulatum s.s.*, *H. capsulatum* LAm A1 (= *H. suramericanum*), *H. capsulatum* LAm B2, and *H. capsulatum* var. *duboisii* (BLAST similarity values >98.6%; Appendix Table 4).

BLAST-based identification of the 40 corresponding COI sequences (see Table in main text) indicated that they represent bat species mainly distributed in Europe from, at least, three genera: *Myotis blythii*, also known as "lesser mouse-eared bat," and congeneric species (83.3%), *Rhinolophus ferrumequinum* or "greater horseshoe bat" (7.14%), and *Miniopterus schreibersii* or "Schreiber's bent-winged bat" (4.76%).

Histoplasma has previously been detected in guano samples of *M. schreibersii* from caves in New South Wales and Australia (*13*). However, this is the first time that *R. ferrumequinum* is associated with *Histoplasma*, previously isolated from the guano of a single species of the same genus, *R. luctus*, in a Malaysian cave (*14*). It is also associated for the first time with *M. blythii*, previously isolated from guano samples of two congeneric bat species, *M. chinensis* and *M. muricola* (*15*). For two *Histoplasma*-positive samples (H10 and H31) we were unable to determine the identity of the source bat species, as we could not amplify the COI gene (Table).

References

1. La_Gaceta_de_Salamanca. Éxito confirmado del Camino de Hierro. 2024 [cited 2024 Oct 12]. https://www.lagacetadesalamanca.es/provincia/exito-confirmado-camino-hierro-20240508122802-nt.html

2. Revilla-Martín N, Alonso-Alonso P, Sereno-Cadierno J, Llanos-Guerrero C, Hernández-Tabernero L, Lizana-Avia M. Bat conservation and ecotourism: the case of two abandoned tunnels in Salamanca, Western Iberia. Barbastella (Alcala Hen). 2021;14:63–8. https://doi.org/10.14709/BarbJ.14.1.2021.09

3. Bialek R, Feucht A, Aepinus C, Just-Nübling G, Robertson VJ, Knobloch J, et al. Evaluation of two nested PCR assays for detection of *Histoplasma capsulatum* DNA in human tissue. J Clin Microbiol. 2002;40:1644–7. PubMed https://doi.org/10.1128/JCM.40.5.1644-1647.2002

4. Kearse M, Moir R, Wilson A, Stones-Havas S, Cheung M, Sturrock S, et al. Geneious Basic: an integrated and extendable desktop software platform for the organization and analysis of sequence data. Bioinformatics. 2012;28:1647–9. PubMed https://doi.org/10.1093/bioinformatics/bts199

5. Kandemir H, Dukik K, de Melo Teixeira M, Stielow JB, Delma FZ, Al-Hatmi A, et al. Phylogenetic and ecological reevaluation of the order Onygenales. Fungal Divers. 2022;115:1–72. https://doi.org/10.1007/s13225-022-00506-z

6. Katoh K, Standley DM. MAFFT multiple sequence alignment software version 7: improvements in performance and usability. Mol Biol Evol. 2013;30:772–80. PubMed https://doi.org/10.1093/molbev/mst010

7. Nguyen L-T, Schmidt HA, von Haeseler A, Minh BQ. IQ-TREE: a fast and effective stochastic algorithm for estimating maximum-likelihood phylogenies. Mol Biol Evol. 2015;32:268–74. PubMed https://doi.org/10.1093/molbev/msu300

8. Kalyaanamoorthy S, Minh BQ, Wong TKF, von Haeseler A, Jermiin LS. ModelFinder: fast model selection for accurate phylogenetic estimates. Nat Methods. 2017;14:587–9. PubMed https://doi.org/10.1038/nmeth.4285

9. Huelsenbeck JP, Ronquist F. MRBAYES: Bayesian inference of phylogenetic trees. Bioinformatics. 2001;17:754–5. PubMed https://doi.org/10.1093/bioinformatics/17.8.754

10. Huelsenbeck JP, Larget B, Alfaro ME. Bayesian phylogenetic model selection using reversible jump Markov chain Monte Carlo. Mol Biol Evol. 2004;21:1123–33. PubMed https://doi.org/10.1093/molbev/msh123

11. Rambaut A, Drummond AJ, Xie D, Baele G, Suchard MA. Posterior summarization in Bayesian phylogenetics using Tracer 1.7. Syst Biol. 2018;67:901–4. PubMed https://doi.org/10.1093/sysbio/syy032

12. Rambaut A. FigTree v 1.4.4. 2018 [cited 2024 Jan 1]. https://github.com/rambaut/figtree/releases/tag/v1.4.4

13. Hunt PJ, Harden TJ, Hibbins M, Pritchard RC, Muir DB, Gardner FJ. *Histoplasma capsulatum*. Isolation from an Australian cave environment and from a patient. Med J Aust. 1984;141:280–3. PubMed https://doi.org/10.5694/j.1326-5377.1984.tb113099.x

14. Ponnampalam J. Isolation of *Histoplasma capsulatum* from the soil of a cave in central Malaysia. Am J Trop Med Hyg. 1963;12:775–6. PubMed https://doi.org/10.4269/ajtmh.1963.12.775

15. Gugnani HC, Denning DW. Infection of bats with *Histoplasma* species. Med Mycol. 2023;61:myad080. PubMed https://doi.org/10.1093/mmy/myad080

16. Walker FM, Williamson CH, Sanchez DE, Sobek CJ, Chambers CL. Species from feces: order-wide identification of Chiroptera from guano and other non-invasive genetic samples. PLoS One. 2016;11:e0162342. PubMed https://doi.org/10.1371/journal.pone.0162342

17. Sepúlveda VE, Márquez R, Turissini DA, Goldman WE, Matute DR. Genome sequences reveal cryptic speciation in the human pathogen *Histoplasma capsulatum.* mBio. 2017;8:e01339–17. PubMed

Appendix Table 1. Data on the 101 DNA extractions from guano samples collected in Camino de Hierro.

DNA extraction number	Collection date	DNA extraction kit	A260/A280 ratio	A260/A230 ratio	Concentration (ng/μL)	Isolate code
1	2023 Feb 27	NZY_s	1.88	1.51	33.5	
2	2023 Feb 27	NZY_s	1.945	1.005	14.9	
3	2023 Feb 27	NZY_s	2	2.015	36	
4	2023 Feb 27	NZY_s	1.93	1.97	45.55	
5	2023 Mar 27	NZY_s	1.8	1.985	48.65	
6	2023 Mar 27	NZY_s	1.835	0.98	14.6	
7	2023 Feb 27	NZY_s	1.805	0.195	10.4	
8	2023 Jan 23	PCI + NZY_t	1.82	1.72	34.6	
9	2023 Jan 23	PCI + NZY_t	1.825	1.525	12.5	
10	2023 Jan 23	PCI + NZY_t	1.81	1.835	13.6	
11	2024 Apr 18	$QIAGEN_{PS}$	1.885	1.675	192.65	
12*	2023 Feb 27	$QIAGEN_{PS}$	1.87	1.295	45.65	H1
13*	2023 Oct 27	$QIAGEN_{PS}$	2.165	0.06	11.65	H2
14	2024 Apr 18	$QIAGEN_{PS}$	1.82	0.568	48	
15	2024 Apr 18	$QIAGEN_{PS}$	1.935	1.045	21.3	
16	2024 Apr 18	$QIAGEN_{PS}$	1.945	0.725	24.55	
17	2024 Apr 18	$QIAGEN_{PS}$	1.91	0.815	23.95	
18	2024 Apr 18	$QIAGEN_{PS}$	1.85	0.93	17	
19	2024 Apr 18	$QIAGEN_{PS}$	2.04	0.96	28.9	
20	2024 Apr 18	$QIAGEN_{PS}$	2.045	0.445	19.4	
21	2024 Apr 18	$QIAGEN_{PS}$	1.935	1.9	100.45	
22*	2024 Apr 18	$QIAGEN_{PS}$	2	0.695	10.35	H3
23	2024 Apr 18	$QIAGEN_{PS}$	1.86	2.09	221.15	
24	2024 Apr 18	$QIAGEN_{PS}$	1.185	0.61	18.55	
25*	2024 Apr 18	$QIAGEN_{PS}$	1.83	1.455	117.65	H4
26	2024 Apr 18	$QIAGEN_{PS}$	1.87	0.705	173.1	
27	2024 Apr 18	$QIAGEN_{PS}$	1.865	1.2	139.5	
28	2024 Apr 18	$QIAGEN_{PS}$	1.95	1.935	101.5	
29	2024 Apr 18	$QIAGEN_{PS}$	1.92	2.03	91.7	
30*	2024 Apr 18	$QIAGEN_{PS}$	1.9	2.325	146.5	H5
31*	2024 Apr 18	$QIAGEN_{PS}$	1.895	1.48	99.95	H6
32	2024 Apr 18	$QIAGEN_{PS}$	1.865	2.22	160.05	
33*	2024 Apr 18	$QIAGEN_{PS}$	2.07	0.84	58.15	H7
34*	2024 Apr 18	$QIAGEN_{PS}$	1.9	0.81	146.3	H8
35*	2024 Apr 18	$QIAGEN_{PS}$	1.895	2.005	76.85	H9
36*	2024 Apr 18	$QIAGEN_{PS}$	1.91	1.545	77.8	H10
37	2024 Apr 18	$QIAGEN_{PS}$	1.855	1.275	101.55	
38*	2024 Apr 18	$QIAGEN_{PS}$	1.89	1.12	62.35	H11

DNA extraction number	Collection date	DNA extraction kit	A260/A280 ratio	A260/A230 ratio	Concentration (ng/μL)	Isolate code
39*	2024 Apr 18	$QIAGEN_{PS}$	1.83	1.48	62.5	H12
40	2024 Apr 18	$QIAGEN_{PS}$	1.86	1.275	64	
41*	2024 Apr 18	$QIAGEN_{PS}$	1.93	1.29	134.45	H13
42	2024 Apr 18	$QIAGEN_{PS}$	1.915	0.35	21.4	
43*	2024 Apr 18	$QIAGEN_{PS}$	1.905	0.29	110.2	H14
44*	2024 Apr 18	$QIAGEN_{PS}$	1.89	1.76	123.05	H15
45*	2024 Apr 18	$QIAGEN_{PS}$	1.885	1.965	95.95	H16
46*	2024 Apr 18	$QIAGEN_{PS}$	1.975	0.71	48.55	H17
47*	2024 Apr 18	$QIAGEN_{PS}$	1.96	0.695	139.95	H18
48*	2023 Feb 27	$QIAGEN_{PS}$	1.635	0.405	6.7	H19
49*	2023 Apr 27	$QIAGEN_{PS}$	1.48	0.27	29.15	H21
50*	2023 Feb 27	$QIAGEN_{PS}$	1.68	0.575	25.9	H22
51*	2023 Feb 27	$QIAGEN_{PS}$	1.74	0.585	20.85	H23
52	2024 Apr 18	$QIAGEN_{PS}$	1.775	0.44	36.25	
53*	2024 Apr 18	$QIAGEN_{PS}$	1.325	0.1	12.25	H26
54	2024 Apr 18	$QIAGEN_{PS}$	1.61	0.695	27.75	
55	2024 Apr 18	$QIAGEN_{PS}$	1.755	0.81	24.75	
56	2024 Apr 18	$QIAGEN_{PS}$	1.4	0.055	6.35	
57	2024 Apr 18	$QIAGEN_{PS}$	1.285	0.295	16.95	
58	2024 Apr 18	$QIAGEN_{PS}$	1.76	0.665	31.65	
59	2024 Apr 18	$QIAGEN_{PS}$	1.495	0.285	22.6	
60	2024 Apr 18	$QIAGEN_{PS}$	0.905	0.135	4.8	
61	2024 Apr 18	$QIAGEN_{PS}$	2.56	0.115	2.9	
62*	2024 Apr 18	$QIAGEN_{PS}$	1.76	1.085	37.5	H27
63	2024 Apr 18	$QIAGEN_{PS}$	1.775	1.17	61.95	
64	2024 Apr 18	$QIAGEN_{PS}$	2.27	0.72	23.45	
65	2024 Apr 18	$QIAGEN_{PS}$	2.315	0.205	6.9	
66	2024 Apr 18	$QIAGEN_{PS}$	2.445	0.44	10.25	
67	2024 Apr 18	$QIAGEN_{PS}$	2.13	0.775	20.3	
68	2024 Apr 18	$QIAGEN_{PS}$	1.77	1.045	26.05	
69	2024 Apr 18	$QIAGEN_{PS}$	2.375	0.09	4.85	
70	2024 Apr 18	$QIAGEN_{PS}$	0.7	0.7	8.85	
71	2024 Apr 18	$QIAGEN_{PS}$	1.47	1.105	65.5	
72	2024 Apr 18	$QIAGEN_{PS}$	1.695	1.33	69	
73	2024 Apr 18	$QIAGEN_{PS}$	1.645	0.19	18.15	
74	2024 Apr 18	$QIAGEN_{PS}$	1.635	1.37	82.5	
75	2024 Apr 18	$QIAGEN_{PS}$	1.79	1.945	154.9	
76	2024 Apr 18	$QIAGEN_{PS}$	1.505	1.04	52.45	
77	2024 Apr 18	$QIAGEN_{PS}$	1.765	1.75	38.7	
78	2024 Apr 18	$QIAGEN_{PS}$	1.32	0.205	24.6	
79	2024 Apr 18	$QIAGEN_{PS}$	1.56	0.86	47.3	
80	2024 Apr 18	$QIAGEN_{PS}$	1.7	0.835	88.35	
81	2024 Apr 18	$QIAGEN_{PS}$	1.72	0.65	20.7	
82	2024 Apr 18	$QIAGEN_{PS}$	1.215	0.035	3.55	
83*	2024 Apr 18	$QIAGEN_{PS}$	1.37	0.895	13.95	H28
84*	2024 Apr 18	$QIAGEN_{PS}$	1.175	0.45	6.75	H29
85*	2024 Apr 18	$QIAGEN_{PS}$	1.35	0.91	8.15	H30
86*	2024 Apr 18	$QIAGEN_{PS}$	0.755	0.605	2	H31
87*	2024 Apr 18	$QIAGEN_{PS}$	2.32	0.28	6.5	H32
88*	2024 Apr 18	$QIAGEN_{PS}$	1.575	0.28	11.7	H33
89*	2024 Apr 18	$QIAGEN_{PS}$	1.99	0.735	15.15	H34
90*	2024 Apr 18	$QIAGEN_{PS}$	1.515	0.355	6.35	H35
91*	2024 Apr 18	$QIAGEN_{PS}$	1.595	0.29	16.15	H36
92*	2024 Apr 18	$QIAGEN_{PS}$	0.61	0.24	1.95	H37
93*	2024 Apr 18	$QIAGEN_{PS}$	0.525	0.025	0.9	H38
94*	2024 Apr 18	$QIAGEN_{PS}$	2.64	1.365	9.45	H39
95*	2024 Apr 18	$QIAGEN_{PS}$	1.56	0.835	17.75	H40
96*	2024 Apr 18	$QIAGEN_{PS}$	1.76	0.21	9.8	H41
97*	2024 Apr 18	$QIAGEN_{PS}$	1.62	0.29	9.9	H42
98*	2024 Apr 18	$QIAGEN_{PS}$	1.815	0.44	3.05	H43
99*	2024 Apr 18	$QIAGEN_{PS}$	1.79	0.52	13.05	H49
100*	2024 Apr 18	$QIAGEN_{PS}$	1.39	0.46	8.8	H50
101	2024 Apr 18	$QIAGEN_{PS}$	2.155	1.225	18.05	

*Positive for Hcp100 are marked with an asterisk. NZY_s, NZY Soil gDNA Isolation kit; NZY_t, NZY Tissue gDNA Isolation kit; PCI, phenol chloroform isoamyl alcohol; $QIAGEN_{PS}$,Qiagen DNeasy Powersoil Pro kit.

Appendix Table 2. Primers and parameters for PCR amplification of Hcp100 and COI

Primer name and sequence (5′–3′)	Amplicon size (bp)	Cycling parameters				Reference
		Step		Temp.	Time	
Histoplasma: first PCR (Hcp100)						
HcI: GCGTTCCGAGCCTTCCACCTCAAC	391	Initial denaturation		95°C	2 min	(*3*)
HcII: ATGTCCCATCGGGCGCCGTGTAGT		x35	Denaturation	95°C	1 min	
			Annealing	65°C	1 min	
			Extension	72°C	2 min	
		Final extension		72°C	10 min	
Histoplasma: nested PCR (Hcp100)						
HcIII: GAGATCTAGTCGCGGCCAGGTTCA	210	Initial denaturation		95°C	5 min	(*3*)
HcIV: AGGAGAGAACTGTATCGGTGGCTTG		x30	Denaturation	95°C	30 s	
			Annealing/ext.	72°C	1 min	
		Final extension		72°C	5 min	
Bat identification (COI)						
SFF-145f: GTHACHGCYCAYGCHTTYGTAATAAT	202	Initial denaturation		95°C	2 min	(*16*)
SFF-351r: TCCWGCRTGDGCWAGRTTTCC		x35	Denaturation	95°C	1 min	
			Annealing	65°C	1 min	
			Extension	72°C	2 min	
		Final extension		72°C	10 min	

Appendix Table 3. Set of 304 Hcp100 sequences originally downloaded from GenBank.

Species name* (genetic group)†	Isolate or strain code	GenBank accession no.‡	Source or host§	Geographic origin
Blastomyces dermatitidis	ER-3	XM_045419905‡	NA	NA
Blastomyces gilchristii	SLH14081	XM_002628281‡	NA	NA
Blastomyces parvus	005–2002	MG544852‡	Clinical sample	Argentina
H. ohiense*	G127B	AJ005963‡	Lab mouse (R)	NA
H. capsulatum s.l.	DMic 02426	KX823346	Clinical sample	Argentina
H. capsulatum s.l.	13123	KX823347	Calomys musculinus (R)	Argentina
H. capsulatum s.l.	17726	KX823348	Calomys musculinus (R)	Argentina
H. capsulatum s.l.	16978	KX823349	Monodelphis dimidiata (M)	Argentina
H. capsulatum s.l.	17679	KX823350	Akodon azarae (R)	Argentina
H. capsulatum s.l.	10174	KX823351‡	Didelphis albiventris (M)	Argentina
H. capsulatum s.l.	13647	KX823352	Calomys laucha (R)	Argentina
H. capsulatum s.l.	13191	KX823353‡	Calomys laucha (R)	Argentina
H. mississippiense*	NAm1	XM_001543550	NA	NA
H. capsulatum s.s.*	G186AR[H]	XM_045433674	NA	Panama
H. capsulatum s.l.	NA	MG913164‡	Vampyriscus bidens (Ch)	Brazil
H. capsulatum s.l.	53465	MW911367‡	Clinical sample	Brazil
H. capsulatum s.l.	53505	MW911368	Clinical sample	Brazil
H. capsulatum s.l.	10ago1101	MF801604‡	Biofertilizers	Colombia
H. capsulatum s.l.	M027	MF801605	Biofertilizers	Colombia
H. capsulatum s.l.	CUEVA100	MF801606	Biofertilizers	Colombia
H. capsulatum s.l.	M025	MF801607	Biofertilizers	Colombia
H. capsulatum s.l.	18nov1006	MF801608	Biofertilizers	Colombia
H. capsulatum s.l.	M014	MF801609	Biofertilizers	Colombia
H. capsulatum s.l.	8nov1101	MF801610	Biofertilizers	Colombia
H. capsulatum s.l.	28feb1203	MF801611	Biofertilizers	Colombia
H. capsulatum s.l.	M030	MF801612	Biofertilizers	Colombia
H. capsulatum s.l.	nn6. FacM	MF801613	Biofertilizers	Colombia
H. capsulatum s.l.	13525	MF801614	Biofertilizers	Colombia
H. capsulatum s.l.	21jul1105	MF801615	Biofertilizers	Colombia
H. capsulatum s.l.	M022	MF801616‡	Biofertilizers	Colombia
H. capsulatum s.l.	R3. 10cm	MF801617	Biofertilizers	Colombia
H. capsulatum s.l.	R3. sup	MF801618	Biofertilizers	Colombia
H. capsulatum s.l.	R3. 15cm	MF801619	Biofertilizers	Colombia
H. capsulatum s.l.	COL_H_020	MH122794‡	Clinical sample	Colombia
H. capsulatum s.l. (LAm B1)	COL_H_037	MH122795	Clinical sample	Colombia
H. capsulatum s.l. (LAm B1)	COL_H_043	MH122796	Clinical sample	Colombia
H. capsulatum s.l. (LAm B1)	COL_H_066	MH122797	Clinical sample	Colombia
H. capsulatum s.l. (LAm B1)	COL_H_036	MH122798	Clinical sample	Colombia
H. capsulatum s.l. (LAm B1)	COL_H_007	MH122799	Clinical sample	Colombia
H. capsulatum s.l. (LAm B1)	COL_H_057	MH122800	Clinical sample	Colombia
H. capsulatum s.l. (LAm B1)	COL_H_008	MH122801	Clinical sample	Colombia
H. capsulatum s.l. (LAm B1)	COL_H_006	MH122802‡	Clinical sample	Colombia
H. capsulatum s.l. (LAm B1)	COL_H_012	MH122803	Clinical sample	Colombia

Species name* (genetic group)†	Isolate or strain code	GenBank accession no.‡	Source or host§	Geographic origin
H. capsulatum s.l. (LAm B1)	COL_H_016	MH122804	Clinical sample	Colombia
H. capsulatum s.l. (LAm B1)	COL_H_017	MH122805	Clinical sample	Colombia
H. capsulatum s.l. (LAm B1)	COL_H_018	MH122806	Clinical sample	Colombia
H. capsulatum s.l. (LAm B1)	COL_H_029	MH122807	Clinical sample	Colombia
H. capsulatum s.l. (LAm B1)	COL_H_032	MH122808	Clinical sample	Colombia
H. capsulatum s.l. (LAm B1)	COL_H_035	MH122809	Clinical sample	Colombia
H. capsulatum s.l. (LAm B1)	COL_H_040	MH122810	Clinical sample	Colombia
H. capsulatum s.l. (LAm B1)	COL_H_056	MH122811	Clinical sample	Colombia
H. capsulatum s.l. (LAm B1)	COL_H_042	MH122812	Clinical sample	Colombia
H. suramericanum* (LAm A2)	COL_H_014	MH122813‡	Clinical sample	Colombia
H. suramericanum* (LAm A1)	COL_H_015	MH122814	Clinical sample	Colombia
H. suramericanum* (LAm A1)	COL_H_024	MH122815	Clinical sample	Colombia
H. suramericanum* (LAm A1)	COL_H_004	MH122816‡	Clinical sample	Colombia
H. suramericanum* (LAm A1)	COL_H_048	MH122817	Clinical sample	Colombia
H. ohiense*	COL_H_001	MH122818‡	Clinical sample	Colombia
H. ohiense*	COL_H_005	MH122819	Clinical sample	Colombia
H. ohiense*	COL_H_038	MH122820	Clinical sample	Colombia
H. suramericanum* (LAm A2)	COL_H_013	MH122821‡	Clinical sample	Colombia
H. suramericanum* (LAm A2)	COL_H_025	MH122822	Clinical sample	Colombia
H. capsulatum s.l.	COL_H_033	MH122823	Clinical sample	Colombia
H. capsulatum s.l.	COL_H_034	MH122824	Clinical sample	Colombia
H. suramericanum* (LAm A2)	COL_H_041	MH122825	Clinical sample	Colombia
H. suramericanum* (LAm A2)	COL_H_055	MH122826	Clinical sample	Colombia
H. suramericanum* (LAm A2)	COL_H_062	MH122827	Clinical sample	Colombia
H. suramericanum* (LAm A1)	COL_H_039	MH122828‡	Clinical sample	Colombia
H. suramericanum* (LAm A2)	COL_H_053	MH122829	Clinical sample	Colombia
H. suramericanum* (LAm A1)	COL_H_019	MH122830‡	Clinical sample	Colombia
H. suramericanum* (LAm A1)	COL_H_021	MH122831	Clinical sample	Colombia
H. suramericanum* (LAm A1)	COL_H_044	MH122832	Clinical sample	Colombia
H. capsulatum (LAm B1)	COL_H_047	MH122833	Clinical sample	Colombia
H. capsulatum (LAm B1)	COL_H_064	MH122834	Clinical sample	Colombia
H. suramericanum* (LAm A1)	COL_S1	MH122835	Chicken manure	Colombia
H. suramericanum* (LAm A1)	COL_S2	MH122836	Chicken manure	Colombia
H. suramericanum* (LAm A1)	COL_S3	MH122837	Chicken manure	Colombia
H. suramericanum* (LAm A2)	COL_H_068	MH122838	Clinical sample	Colombia
H. capsulatum s.l.	M-431P	JF270313	Tadarida brasiliensis (Ch)	Mexico
H. capsulatum s.l.	M-433P	JF270314	Tadarida brasiliensis (Ch)	Mexico
H. capsulatum s.l.	M-434P	JF270315	Tadarida brasiliensis (Ch)	Mexico
H. capsulatum s.l.	M-435P	JF270316	Tadarida brasiliensis (Ch)	Mexico
H. capsulatum s.l.	M-436P	JF270317	Tadarida brasiliensis (Ch)	Mexico
H. capsulatum s.l.	M-441P	JF270318	Tadarida brasiliensis (Ch)	Mexico
H. capsulatum s.l.	M-444P	JF270319	Tadarida brasiliensis (Ch)	Mexico
H. capsulatum s.l.	M-445P	JF270320‡	Tadarida brasiliensis (Ch)	Mexico
H. capsulatum s.l.	M-446P	JF270321	Tadarida brasiliensis (Ch)	Mexico
H. capsulatum s.l.	M-447P	JF270322	Tadarida brasiliensis (Ch)	Mexico
H. capsulatum s.l.	M-448P	JF270323	Tadarida brasiliensis (Ch)	Mexico
H. capsulatum s.l.	M-453P	JF270324	Tadarida brasiliensis (Ch)	Mexico
H. capsulatum s.l.	M-455P	JF270325	Tadarida brasiliensis (Ch)	Mexico
H. capsulatum s.l.	M-456P	JF270326	Tadarida brasiliensis (Ch)	Mexico
H. capsulatum s.l.	M-457P	JF270327	Tadarida brasiliensis (Ch)	Mexico
H. capsulatum s.l.	M-458P	JF270328	Tadarida brasiliensis (Ch)	Mexico
H. capsulatum s.l.	M-459P	JF270329	Tadarida brasiliensis (Ch)	Mexico
H. capsulatum s.l.	M-460P	JF270330	Tadarida brasiliensis (Ch)	Mexico
H. capsulatum s.l.	M-462P	JF270331	Tadarida brasiliensis (Ch)	Mexico
H. capsulatum s.l.	M-463P	JF270332	Tadarida brasiliensis (Ch)	Mexico
H. capsulatum s.l.	M-468P	JF270333	Tadarida brasiliensis (Ch)	Mexico
H. capsulatum s.l.	M-469P	JF270334	Tadarida brasiliensis (Ch)	Mexico
H. capsulatum s.l.	M-470P	JF270335	Tadarida brasiliensis (Ch)	Mexico
H. capsulatum s.l.	M-471P	JF270336	Tadarida brasiliensis (Ch)	Mexico
H. capsulatum s.l.	M-473P	JF270337	Tadarida brasiliensis (Ch)	Mexico
H. capsulatum s.l.	M-474P	JF270338	Tadarida brasiliensis (Ch)	Mexico
H. capsulatum s.l.	M-475P	JF270339	Tadarida brasiliensis (Ch)	Mexico
H. capsulatum s.l.	M-476P	JF270340	Tadarida brasiliensis (Ch)	Mexico
H. capsulatum s.l.	M-477P	JF270341	Tadarida brasiliensis (Ch)	Mexico
H. capsulatum s.l.	M-478P	JF270342	Tadarida brasiliensis (Ch)	Mexico
H. capsulatum s.l.	M-479P	JF270343	Tadarida brasiliensis (Ch)	Mexico
H. capsulatum s.l.	M-480P	JF270344	Tadarida brasiliensis (Ch)	Mexico
H. capsulatum s.l.	M-481P	JF270345	Tadarida brasiliensis (Ch)	Mexico
H. capsulatum s.l.	M-482P	JF270346	Tadarida brasiliensis (Ch)	Mexico

Species name* (genetic group)†	Isolate or strain code	GenBank accession no.‡	Source or host§	Geographic origin
H. capsulatum s.l.	M-483P	JF270347	Tadarida brasiliensis (Ch)	Mexico
H. capsulatum s.l.	M-485P	JF270348	Tadarida brasiliensis (Ch)	Mexico
H. capsulatum s.l.	M-486P	JF270349	Tadarida brasiliensis (Ch)	Mexico
H. capsulatum s.l.	M-487P	JF270350	Tadarida brasiliensis (Ch)	Mexico
H. capsulatum s.l.	M-489P	JF270351	Tadarida brasiliensis (Ch)	Mexico
H. capsulatum s.l.	M-490P	JF270352‡	Tadarida brasiliensis (Ch)	Mexico
H. capsulatum s.l.	M-491P	JF270353	Tadarida brasiliensis (Ch)	Mexico
H. capsulatum s.l.	M-492P	JF270354	Tadarida brasiliensis (Ch)	Mexico
H. capsulatum s.l.	M-493P	JF270355	Tadarida brasiliensis (Ch)	Mexico
H. capsulatum s.l.	M-494P	JF270356	Tadarida brasiliensis (Ch)	Mexico
H. capsulatum s.l.	M-495P	JF270357	Tadarida brasiliensis (Ch)	Mexico
H. capsulatum s.l.	M-497P	JF270358	Tadarida brasiliensis (Ch)	Mexico
H. capsulatum s.l.	M-502P	JF270359	Tadarida brasiliensis (Ch)	Mexico
H. capsulatum s.l.	M-503P	JF270360	Tadarida brasiliensis (Ch)	Mexico
H. capsulatum s.l.	M-507P	JF270361	Tadarida brasiliensis (Ch)	Mexico
H. capsulatum s.l.	M-510P	JF270362	Tadarida brasiliensis (Ch)	Argentina
H. capsulatum s.l.	M-511P	JF270363	Tadarida brasiliensis (Ch)	Argentina
H. capsulatum s.l.	M-512P	JF270364	Tadarida brasiliensis (Ch)	Argentina
H. capsulatum s.l.	M-513P	JF270365	Tadarida brasiliensis (Ch)	Argentina
H. capsulatum s.l.	M-514P	JF270366	Tadarida brasiliensis (Ch)	Argentina
H. capsulatum s.l.	M-515P	JF270367	Tadarida brasiliensis (Ch)	Argentina
H. capsulatum s.l.	M-516P	JF270368	Tadarida brasiliensis (Ch)	Argentina
H. capsulatum s.l.	M-517P	JF270369	Tadarida brasiliensis (Ch)	Argentina
H. capsulatum s.l.	M-518P	JF270370	Tadarida brasiliensis (Ch)	Argentina
H. capsulatum s.l.	M-519P	JF270371	Tadarida brasiliensis (Ch)	Argentina
H. capsulatum s.l.	M-520P	JF270372	Tadarida brasiliensis (Ch)	Argentina
H. capsulatum s.l.	M-521P	JF270373	Tadarida brasiliensis (Ch)	Argentina
H. capsulatum s.l.	M-522P	JF270374	Tadarida brasiliensis (Ch)	Argentina
H. capsulatum s.l.	M-523P	JF270375	Tadarida brasiliensis (Ch)	Argentina
H. capsulatum s.l.	M-524P	JF270376	Tadarida brasiliensis (Ch)	Argentina
H. capsulatum s.l.	M-525P	JF270377	Tadarida brasiliensis (Ch)	Argentina
H. capsulatum s.l.	M-AR01P	JF270378	Tadarida brasiliensis (Ch)	Argentina
H. capsulatum s.l.	M-AR03P	JF270379‡	Tadarida brasiliensis (Ch)	Argentina
H. capsulatum s.l.	M-AR05P	JF270380	Tadarida brasiliensis (Ch)	Argentina
H. capsulatum s.l.	M-484P	HM921044	Tadarida brasiliensis (Ch)	Mexico
H. capsulatum s.l.	M-501P	HM921045	Tadarida brasiliensis (Ch)	Mexico
H. capsulatum s.l.	M-506P	HM921046	Tadarida brasiliensis (Ch)	Mexico
H. capsulatum s.l.	M-527P	HM921047	Glossophaga soricina (Ch)	Mexico
H. capsulatum s.l.	N014	HM921048	Nyctalus noctula (Ch)	France
H. capsulatum s.l.	M-420	JX091346	Artibeus hirsutus (Ch)	Mexico
H. capsulatum s.l.	M-421	JX091347	Artibeus hirsutus (Ch)	Mexico
H. capsulatum s.l.	M-422	JX091348	Artibeus hirsutus (Ch)	Mexico
H. capsulatum s.l.	M-425	JX091349	Artibeus hirsutus (Ch)	Mexico
H. capsulatum s.l.	M-426	JX091350	Artibeus hirsutus (Ch)	Mexico
H. capsulatum s.l.	M-466P	JX091351	Mormoops megalophylla (Ch)	Mexico
H. capsulatum s.l.	M-467P	JX091352	Myotis californicus (Ch)	Mexico
H. capsulatum s.l.	M-526P	JX091353	Glossophaga sp. (Ch)	Mexico
H. capsulatum s.l.	M-527P	JX091354	Glossophaga sp. (Ch)	Mexico
H. capsulatum s.l.	M-528P	JX091355	Glossophaga sp. (Ch)	Mexico
H. capsulatum s.l.	M-529P	JX091356	Glossophaga sp. (Ch)	Mexico
H. capsulatum s.l.	M-530P	JX091357	Natalus stramineus (Ch)	Mexico
H. capsulatum s.l.	M-531P	JX091358	Natalus stramineus (Ch)	Mexico
H. capsulatum s.l.	M-535P	JX091359	Natalus stramineus (Ch)	Mexico
H. capsulatum s.l.	M-536P	JX091360	Natalus stramineus (Ch)	Mexico
H. capsulatum s.l.	M-537P	JX091361	Natalus stramineus (Ch)	Mexico
H. capsulatum s.l.	M-538P	JX091362	Mormoops megalophylla (Ch)	Mexico
H. capsulatum s.l.	M-539P	JX091363	Mormoops megalophylla (Ch)	Mexico
H. capsulatum s.l.	M-540P	JX091364	Pteronotus davyi (Ch)	Mexico
H. capsulatum s.l.	M-541P	JX091365	Pteronotus parnellii (Ch)	Mexico
H. capsulatum s.l.	M-542P	JX091366	Pteronotus parnellii (Ch)	Mexico
H. capsulatum s.l.	G-8P	JX091367	Glossophaga soricina (Ch)	French Guiana
H. capsulatum s.l.	G-12P	JX091368‡	Glossophaga soricina (Ch)	French Guiana
H. capsulatum s.l.	G-13P	JX091369	Carollia perspicillata (Ch)	French Guiana
H. capsulatum s.l.	G-18P	JX091370	Glossophaga soricina (Ch)	French Guiana
H. capsulatum s.l.	M-421B	JX138902‡	Artibeus hirsutus (Ch)	Mexico
H. capsulatum s.l.	M-422B	JX138903	Artibeus hirsutus (Ch)	Mexico

Species name* (genetic group)†	Isolate or strain code	GenBank accession no.‡	Source or host§	Geographic origin
H. capsulatum s.l.	M-425B	JX138904	Artibeus hirsutus (Ch)	Mexico
H. capsulatum s.l.	M-426B	JX138905	Artibeus hirsutus (Ch)	Mexico
H. capsulatum s.l.	M-444B	JX138906	Tadarida brasiliensis (Ch)	Mexico
H. capsulatum s.l.	M-445B	JX138907	Tadarida brasiliensis (Ch)	Mexico
H. capsulatum s.l.	M-446B	JX138908	Tadarida brasiliensis (Ch)	Mexico
H. capsulatum s.l.	M-450B	JX138909	Tadarida brasiliensis (Ch)	Mexico
H. capsulatum s.l.	M-453B	JX138910	Tadarida brasiliensis (Ch)	Mexico
H. capsulatum s.l.	M-454B	JX138911	Tadarida brasiliensis (Ch)	Mexico
H. capsulatum s.l.	M-455B	JX138912	Tadarida brasiliensis (Ch)	Mexico
H. capsulatum s.l.	M-456B	JX138913	Tadarida brasiliensis (Ch)	Mexico
H. capsulatum s.l.	M-458B	JX138914	Tadarida brasiliensis (Ch)	Mexico
H. capsulatum s.l.	M-459B	JX138915	Tadarida brasiliensis (Ch)	Mexico
H. capsulatum s.l.	M-460B	JX138916	Tadarida brasiliensis (Ch)	Mexico
H. capsulatum s.l.	M-468B	JX138917	Tadarida brasiliensis (Ch)	Mexico
H. capsulatum s.l.	M-470B	JX138918	Tadarida brasiliensis (Ch)	Mexico
H. capsulatum s.l.	M-471B	JX138919	Tadarida brasiliensis (Ch)	Mexico
H. capsulatum s.l.	M-473B	JX138920	Tadarida brasiliensis (Ch)	Mexico
H. capsulatum s.l.	M-474B	JX138921	Tadarida brasiliensis (Ch)	Mexico
H. capsulatum s.l.	M-476B	JX138922	Tadarida brasiliensis (Ch)	Mexico
H. capsulatum s.l.	M-478B	JX138923	Tadarida brasiliensis (Ch)	Mexico
H. capsulatum s.l.	M-480B	JX138924	Tadarida brasiliensis (Ch)	Mexico
H. capsulatum s.l.	M-481B	JX138925	Tadarida brasiliensis (Ch)	Mexico
H. capsulatum s.l.	M-483B	JX138926	Tadarida brasiliensis (Ch)	Mexico
H. capsulatum s.l.	M-484B	JX138927	Tadarida brasiliensis (Ch)	Mexico
H. capsulatum s.l.	M-485B	JX138928	Tadarida brasiliensis (Ch)	Mexico
H. capsulatum s.l.	M-487B	JX138929	Tadarida brasiliensis (Ch)	Mexico
H. capsulatum s.l.	M-501B	JX138930	Tadarida brasiliensis (Ch)	Mexico
H. capsulatum s.l.	M-506B	JX138931	Tadarida brasiliensis (Ch)	Mexico
H. capsulatum s.l.	M-507B	JX138932	Tadarida brasiliensis (Ch)	Mexico
H. capsulatum s.l.	M-510B	JX138933	Tadarida brasiliensis (Ch)	Argentina
H. capsulatum s.l.	M-511B	JX138934	Tadarida brasiliensis (Ch)	Argentina
H. capsulatum s.l.	M-512B	JX138935	Tadarida brasiliensis (Ch)	Argentina
H. capsulatum s.l.	M-513B	JX138936	Tadarida brasiliensis (Ch)	Argentina
H. capsulatum s.l.	M-514B	JX138937	Tadarida brasiliensis (Ch)	Argentina
H. capsulatum s.l.	M-515B	JX138938	Tadarida brasiliensis (Ch)	Argentina
H. capsulatum s.l.	M-516B	JX138939	Tadarida brasiliensis (Ch)	Argentina
H. capsulatum s.l.	M-517B	JX138940	Tadarida brasiliensis (Ch)	Argentina
H. capsulatum s.l.	M-518B	JX138941	Tadarida brasiliensis (Ch)	Argentina
H. capsulatum s.l.	M-519B	JX138942	Tadarida brasiliensis (Ch)	Argentina
H. capsulatum s.l.	M-520B	JX138943	Tadarida brasiliensis (Ch)	Argentina
H. capsulatum s.l.	M-521B	JX138944	Tadarida brasiliensis (Ch)	Argentina
H. capsulatum s.l.	M-522B	JX138945	Tadarida brasiliensis (Ch)	Argentina
H. capsulatum s.l.	M-523B	JX138946	Tadarida brasiliensis (Ch)	Argentina
H. capsulatum s.l.	M-524B	JX138947	Tadarida brasiliensis (Ch)	Argentina
H. capsulatum s.l.	M-525B	JX138948	Tadarida brasiliensis (Ch)	Argentina
H. capsulatum s.l.	M-526B	JX138949	Glossophaga sp. (Ch)	Mexico
H. capsulatum s.l.	M-527B	JX138950	Glossophaga sp. (Ch)	Mexico
H. capsulatum s.l.	M-528B	JX138951	Glossophaga sp. (Ch)	Mexico
H. capsulatum s.l.	M-529B	JX138952	Glossophaga sp. (Ch)	Mexico
H. capsulatum s.l.	M-530B	JX138953	Natalus stramineus (Ch)	Mexico
H. capsulatum s.l.	M-531B	JX138954	Natalus stramineus (Ch)	Mexico
H. capsulatum s.l.	M-532B	JX138955	Natalus stramineus (Ch)	Mexico
H. capsulatum s.l.	M-535B	JX138956	Natalus stramineus (Ch)	Mexico
H. capsulatum s.l.	M-536B	JX138957	Natalus stramineus (Ch)	Mexico
H. capsulatum s.l.	M-538B	JX138958	Mormoops megalophylla (Ch)	Mexico
H. capsulatum s.l.	M-539B	JX138959	Mormoops megalophylla (Ch)	Mexico
H. capsulatum s.l.	M-541B	JX138960	Pteronotus parnellii (Ch)	Mexico
H. capsulatum s.l.	M-542B	JX138961	Pteronotus parnellii (Ch)	Mexico
H. capsulatum s.l.	M-AR01	JX138962	Tadarida brasiliensis (Ch)	Argentina
H. capsulatum s.l.	M-AR03	JX138963	Tadarida brasiliensis (Ch)	Argentina
H. capsulatum s.l.	Hc-TYM	LC517841‡	Clinical sample	Japan
H. capsulatum s.l.	c12	KF225552‡	Clinical sample	Cuba
H. capsulatum s.l.	c57	KF225553‡	Clinical sample	Cuba
H. capsulatum s.l.	F49	MZ713369	Bird excreta	Antarctica
H. capsulatum s.l.	F54	MZ713370	Bird excreta	Antarctica
H. capsulatum s.l.	S269	MZ713371	Soil	Antarctica
H. capsulatum s.l.	F47	MZ713372‡	Bird excreta	Antarctica

Species name* (genetic group)†	Isolate or strain code	GenBank accession no.‡	Source or host§	Geographic origin
H. capsulatum s.l.	S268B	MZ713373‡	Soil	Antarctica
H. capsulatum s.l.	03.16	MZ713374‡	Clinical sample	Brazil
H. suramericanum* (RJ)	24.11	MZ713375‡	Clinical sample	Brazil
H. capsulatum s.l.	20231	MZ713376	Clinical sample	Brazil
H. capsulatum s.s.*	39942	MZ713377‡	Clinical sample	Brazil
H. capsulatum s.s.*	G184A	MZ713378‡	Clinical sample	Panama
H. ohiense*	G217B[H]	MZ713379‡	Clinical sample	USA
H. capsulatum s.l.	01.16	MZ713380‡	Clinical sample	Brazil
H. capsulatum s.l.	55205	OR242318	Bat guano	Ecuador
H. capsulatum s.l.	55221	OR242319‡	Bat guano	Ecuador
H. capsulatum s.l.	55225	OR242320	Bat guano	Ecuador
H. capsulatum s.l.	55267	OR242321	Bat guano	Ecuador
H. capsulatum s.l.	U003	OR242322	Undetermined species (Ch)	Ecuador
H. capsulatum s.l.	U007	OR242323	Undetermined species (Ch)	Ecuador
H. capsulatum s.l.	U008	OR242324	Undetermined species (Ch)	Ecuador
H. capsulatum s.l.	U009	OR242325	Undetermined species (Ch)	Ecuador
H. capsulatum s.l.	55151	OR242326‡	Undetermined species (Ch)	Ecuador
H. capsulatum s.l.	55161	OR242327‡	Undetermined species (Ch)	Ecuador
H. capsulatum s.l.	55162	OR242328‡	Undetermined species (Ch)	Ecuador
H. capsulatum s.l.	55164	OR242329‡	Undetermined species (Ch)	Ecuador
H. capsulatum s.l.	55165	OR242330	Undetermined species (Ch)	Ecuador
H. capsulatum s.l.	55166	OR242331	Undetermined species (Ch)	Ecuador
H. capsulatum s.l.	55170	OR242332	Undetermined species (Ch)	Ecuador
H. capsulatum s.l.	55174	OR242333‡	Undetermined species (Ch)	Ecuador
H. capsulatum s.l.	55175	OR242334	Undetermined species (Ch)	Ecuador
H. capsulatum s.l.	55176	OR242335‡	Undetermined species (Ch)	Ecuador
H. capsulatum s.l.	55185	OR242336‡	Undetermined species (Ch)	Ecuador
H. capsulatum s.l.	55186	OR242337	Undetermined species (Ch)	Ecuador
H. capsulatum s.l.	55187	OR242338	Undetermined species (Ch)	Ecuador
H. capsulatum s.l.	55189	OR242339	Undetermined species (Ch)	Ecuador
H. capsulatum s.l.	55194	OR242340	Undetermined species (Ch)	Ecuador
H. capsulatum s.l.	55195	OR242341	Undetermined species (Ch)	Ecuador
H. capsulatum s.l.	55202	OR242342	Undetermined species (Ch)	Ecuador
H. capsulatum s.l.	55204	OR242343	Undetermined species (Ch)	Ecuador
H. capsulatum s.l.	55209	OR242344‡	Undetermined species (Ch)	Ecuador
H. capsulatum s.l.	55215	OR242345‡	Undetermined species (Ch)	Ecuador
H. capsulatum s.l.	55216	OR242346	Undetermined species (Ch)	Ecuador
H. capsulatum s.l.	55219	OR242347	Undetermined species (Ch)	Ecuador
H. capsulatum s.l.	55225	OR242348	Undetermined species (Ch)	Ecuador
H. capsulatum s.l.	55233	OR242349	Undetermined species (Ch)	Ecuador
H. capsulatum s.l.	55239	OR242350	Undetermined species (Ch)	Ecuador
H. capsulatum s.l.	55242	OR242351	Undetermined species (Ch)	Ecuador
H. capsulatum s.l.	55252	OR242352	Undetermined species (Ch)	Ecuador
H. capsulatum s.l.	55255	OR242353	Undetermined species (Ch)	Ecuador
H. capsulatum s.l.	55264	OR242354	Undetermined species (Ch)	Ecuador
H. capsulatum s.l.	55281	OR242355	Undetermined species (Ch)	Ecuador
H. mississippiense*	RMSCC1001	KC990358‡	Clinical sample	USA
H. ohiense*	H18	KC990359‡	Clinical sample	USA
H. suramericanum* (LAm A1)	H60	KC990360‡	Clinical sample	Colombia
H. suramericanum* (LAm A2)	H67	KC990361‡	Clinical sample	Colombia
H. capsulatum (LAm B1)	H59	KC990362	Clinical sample	Colombia
H. capsulatum var. duboisii*	H91	KC990363	Clinical sample	Liberia
H. capsulatum s.l.	H176	KC990364‡	Clinical sample	Netherlands
H. capsulatum (LAm B2)	H66	KC990365‡	Clinical sample	Colombia
H. capsulatum (LAm B2)	H69	KC990366‡	Clinical sample	Colombia
H. capsulatum s.s.*	H81	KC990367	Clinical sample	Panama
H. capsulatum var. duboisii	H88	CP069104‡	Clinical sample	Belgium
H. mississippiense*	WU24	CP069109‡	Clinical sample	USA
H. capsulatum s.s.*	G186AR[H]	CP069122‡	Clinical sample	Panama

*Species name according to Sepúlveda *et al.* (*17*). Based on the information available in the literature or GenBank for that specific isolate: *H. capsulatum s.s.* (= *H. capsulatum* Panama or H81 lineage); *H. mississippiense* (= *H. capsulatum* NAm 1); *H. ohiense* (= *H. capsulatum* NAm 2); *H. suramericanum* (= *H. capsulatum* LAm A); and *H. capsulatum* var. *duboisii* (= *H. capsulatum* African lineage or *H. duboisii*). H, holotype; NA, not available.
†Group or sublineage, indicated only when available in previous molecular studies.
‡Unique GenBank sequence included for reference purposes in our phylogenetic analyses.
§Host: (Ch), chiropters; (M), marsupials; (R), rodents.

Appendix Table 4. Hcp100 sequences obtained from guano samples collected in Camino de Hierro and putative identity based on BLAST results.

Seq. ID (accession no.)	Most similar GenBank accessions*	Identity (%)	Species† (genetic sublineage)‡	Country
H1	MH122817	100.00	*H. suramericanum* (LAm A1)	Colombia
(PP887829)	MH122816	100.00	*H. suramericanum* (LAm A1)	Colombia
H2	MH122817	100.00	*H. suramericanum* (LAm A1)	Colombia
(PP887830)	MH122816	100.00	*H. suramericanum* (LAm A1)	Colombia
H3	MH122817	99.49	*H. suramericanum* (LAm A1)	Colombia
(PP887831)	MH122816	99.49	*H. suramericanum* (LAm A1)	Colombia
H4	KC990365	98.57	*H. capsulatum s.l.* (LAm B2)	Colombia
(PP887832)	KC990363	98.57	*H. capsulatum* var. *duboisii*	Liberia
H5	MH122817	100.00	*H. suramericanum* (LAm A1)	Colombia
(PP887833)	MH122816	100.00	*H. suramericanum* (LAm A1)	Colombia
H6	KC990365	99.52	*H. capsulatum s.l.* (LAm B2)	Colombia
(PP887834)	KC990363	99.52	*H. capsulatum* var. *duboisii*	Liberia
H7	KC990365	99.52	*H. capsulatum s.l.* (LAm B2)	Colombia
(PP887835)	KC990363	99.52	*H. capsulatum* var. *duboisii*	Liberia
H8	KC990365	99.52	*H. capsulatum s.l.* (LAm B2)	Colombia
(PP887836)	KC990363	99.52	*H. capsulatum* var. *duboisii*	Liberia
H9	MH122817	100.00	*H. suramericanum* (LAm A1)	Colombia
(PP887837)	MH122816	100.00	*H. suramericanum* (LAm A1)	Colombia
H10	MH122817	99.49	*H. suramericanum* (LAm A1)	Colombia
(PP887838)	MH122816	99.49	*H. suramericanum* (LAm A1)	Colombia
H11	KC990365	99.52	*H. capsulatum s.l.* (LAm B2)	Colombia
(PP887839)	KC990363	99.52	*H. capsulatum* var. *duboisii*	Liberia
H12	MH122817	100.00	*H. suramericanum* (LAm A1)	Colombia
(PP887840)	MH122816	100.00	*H. suramericanum* (LAm A1)	Colombia
H13	MH122817	99.49	*H. suramericanum* (LAm A1)	Colombia
(PP887841)	MH122816	99.49	*H. suramericanum* (LAm A1)	Colombia
H14	MH122817	100.00	*H. suramericanum* (LAm A1)	Colombia
(PP887842)	MH122816	100.00	*H. suramericanum* (LAm A1)	Colombia
H15	MH122817	100.00	*H. suramericanum* (LAm A1)	Colombia
(PP887843)	MH122816	100.00	*H. suramericanum* (LAm A1)	Colombia
H16	MH122817	100.00	*H. suramericanum* (LAm A1)	Colombia
(PP887844)	MH122816	100.00	*H. suramericanum* (LAm A1)	Colombia
H17	KC990365	99.52	*H. capsulatum s.l.* (LAm B2)	Colombia
(PP887845)	KC990363	99.52	*H. capsulatum* var. *duboisii*	Liberia
H18	MH122817	100.00	*H. suramericanum* (LAm A1)	Colombia
(PP887846)	MH122816	100.00	*H. suramericanum* (LAm A1)	Colombia
H19	MH122817	100.00	*H. suramericanum* (LAm A1)	Colombia
(PP887847)	MH122816	100.00	*H. suramericanum* (LAm A1)	Colombia
H21	MH122817	100.00	*H. suramericanum* (LAm A1)	Colombia
(PP887849)	MH122816	100.00	*H. suramericanum* (LAm A1)	Colombia
H22	MH122817	100.00	*H. suramericanum* (LAm A1)	Colombia
(PP887850)	MH122816	100.00	*H. suramericanum* (LAm A1)	Colombia
H23	MH122817	100.00	*H. suramericanum* (LAm A1)	Colombia
(PP887851)	MH122816	100.00	*H. suramericanum* (LAm A1)	Colombia
H26	MH122817	100.00	*H. suramericanum* (LAm A1)	Colombia
(PP887854)	MH122816	100.00	*H. suramericanum* (LAm A1)	Colombia
H27	MH122817	100.00	*H. suramericanum* (LAm A1)	Colombia
(PP887855)	MH122816	100.00	*H. suramericanum* (LAm A1)	Colombia
H28	MH122817	100.00	*H. suramericanum* (LAm A1)	Colombia
(PP887856)	MH122816	100.00	*H. suramericanum* (LAm A1)	Colombia
H29	MH122817	98.97	*H. suramericanum* (LAm A1)	Colombia
(PP887857)	MH122816	98.97	*H. suramericanum* (LAm A1)	Colombia
H30	KC990365	99.52	*H. capsulatum s.l.* (LAm B2)	Colombia
(PP887858)	KC990363	99.52	*H. capsulatum* var. *duboisii*	Liberia
H31	MH122817	99.49	*H. suramericanum* (LAm A1)	Colombia
(PP887859)	MH122816	99.49	*H. suramericanum* (LAm A1)	Colombia
H32	MH122817	99.49	*H. suramericanum* (LAm A1)	Colombia
(PP887860)	MH122816	99.49	*H. suramericanum* (LAm A1)	Colombia
H33	MH122817	100.00	*H. suramericanum* (LAm A1)	Colombia
(PP887861)	MH122816	100.00	*H. suramericanum* (LAm A1)	Colombia
H34	KC990365	99.52	*H. capsulatum s.l.* (LAm B2)	Colombia
(PP887862)	KC990363	99.52	*H. capsulatum* var. *duboisii*	Liberia
H35	KC990365	99.52	*H. capsulatum s.l.* (LAm B2)	Colombia

Seq. ID (accession no.)	Most similar GenBank accessions*	Identity (%)	Species† (genetic sublineage)‡	Country
(PP887863)	KC990363	99.52	*H. capsulatum* var. *duboisii*	Liberia
H36	MH122817	99.49	*H. suramericanum* (LAm A1)	Colombia
(PP887864)	MH122816	99.49	*H. suramericanum* (LAm A1)	Colombia
H37	MZ713377	99.47	*H. capsulatum s.s.*	Brazil
(PP887865)	KC990363	99.06	*H. capsulatum* var. *duboisii*	Liberia
H38	KC990365	99.52	*H. capsulatum s.l.* (LAm B2)	Colombia
(PP887866)	KC990363	99.52	*H. capsulatum* var. *duboisii*	Liberia
H39	KC990365	99.52	*H. capsulatum s.l.* (LAm B2)	Colombia
(PP887867)	KC990363	99.52	*H. capsulatum* var. *duboisii*	Liberia
H40	KC990365	99.52	*H. capsulatum s.l.* (LAm B2)	Colombia
(PP887868)	KC990363	99.52	*H. capsulatum* var. *duboisii*	Liberia
H41	MH122817	100.00	*H. suramericanum* (LAm A1)	Colombia
(PP887869)	MH122816	100.00	*H. suramericanum* (LAm A1)	Colombia
H42	KC990365	100.00	*H. capsulatum s.l.* (LAm B2)	Colombia
(PP887870)	KC990363	100.00	*H. capsulatum* var. *duboisii*	Liberia
H43	MZ713377	100.00	*H. capsulatum s.s.*	Brazil
(PP887871)	KC990365	99.52	*H. capsulatum s.l.* (LAm B2)	Colombia
	KC990363	99.52	*H. capsulatum* var. *duboisii*	Liberia
H49	MH122817	100.00	*H. suramericanum* (LAm A1)	Colombia
(PP887877)	MH122816	100.00	*H. suramericanum* (LAm A1)	Colombia
H50	MH122817	100.00	*H. suramericanum* (LAm A1)	Colombia
(PP887878)	MH122816	100.00	*H. suramericanum* (LAm A1)	Colombia

*For each isolate, at least, two hits with identical or almost identical sequence identity values were found.
†Species name according to Sepúlveda *et al.* (*17*): *H. capsulatum s.s.* (= *H. capsulatum* Panama or H81 lineage); *H. capsulatum* var. *duboisii* (= *H. capsulatum* African lineage or *H. duboisii*); and *H. suramericanum* (= *H. capsulatum* LAm A).
‡ Genetic subgroup or sublineage, indicated only when this information is available in previous molecular studies.

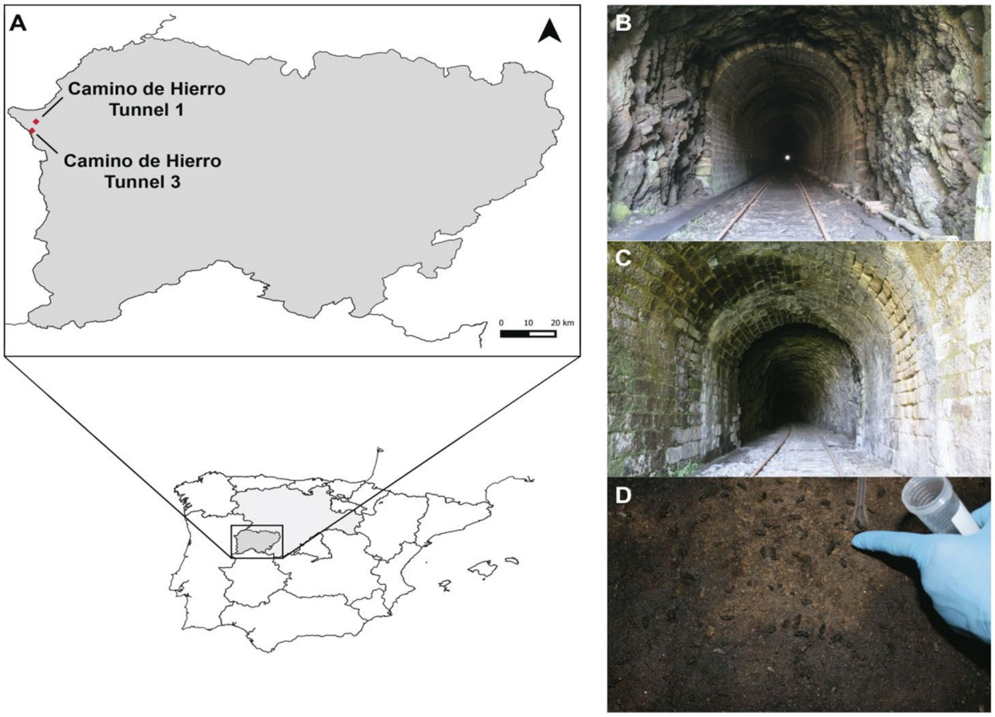

Appendix Figure 1. Sampling sites. A) Map of the Iberian Peninsula showing the approximate geographic location of the sampling points. The province of Salamanca (Castilla y León, Spain) is shaded is dark grey. B) Exit of La Carretera tunnel (tunnel 1). C) Exit of Morgado tunnel (tunnel 3). D) Appearance of the bat feces collected. Note the almost intact rod shape of the excrements, suggesting recent defecation. Photos by Laura Noelly Niño Puerto.

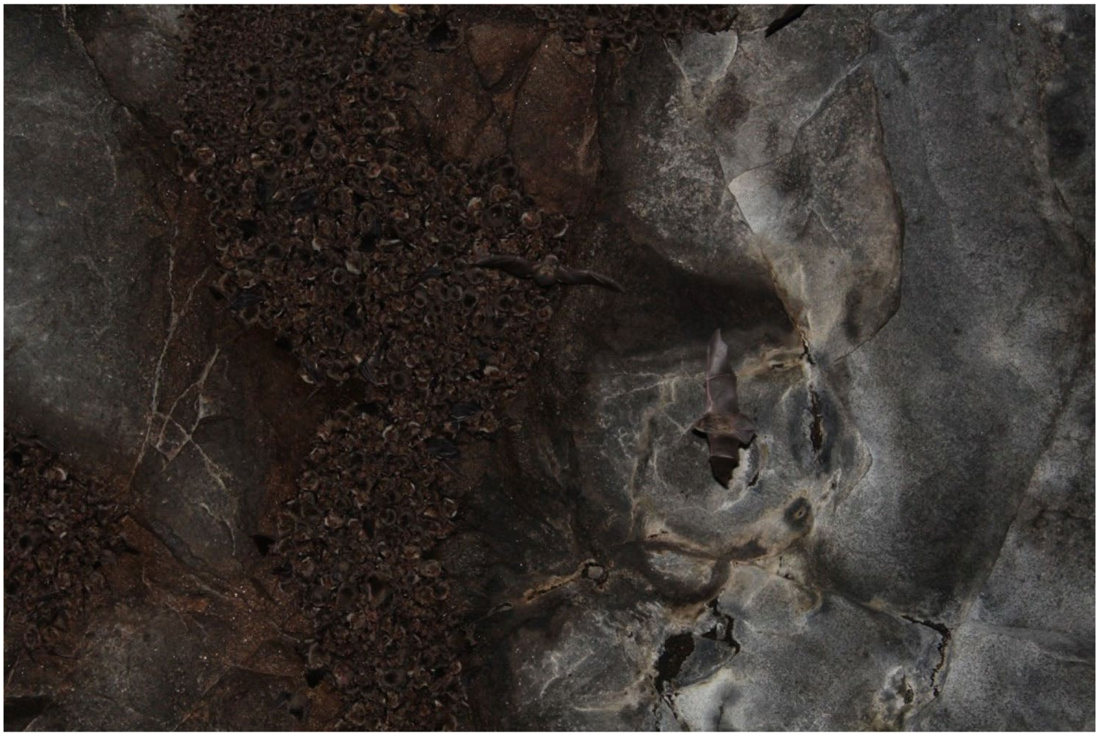

Appendix Figure 2. Small group of bats roosting together in the roof of Morgado tunnel. Photo by Laura Noelly Niño Puerto.
